# A Case of Moyamoya Disease With Spontaneous Regression of Posterior Choroidal Artery Aneurysm After Improvement in Obesity and Administration of an Antihypertensive Drug

**DOI:** 10.7759/cureus.72458

**Published:** 2024-10-26

**Authors:** Akihito Hashiguchi, Takeshi Tonegawa, Kozo Tashima, Shuki Mizukami, Koichi Moroki

**Affiliations:** 1 Neurological Surgery, Tokuda Neurosurgical Hospital, Kanoya, JPN

**Keywords:** choroidal anastomosis, choroidal aneurysm, moyamoya disease (mmd), obesity, spontaneous regression

## Abstract

A 24-year-old obese female (height = 162 cm, weight = 84 kg, and BMI = 32.0) developed transient dysarthria and left hemiparesis, which was diagnosed as moyamoya disease (MMD) after imaging studies. Cerebral angiography and single photon emission computed tomography studies revealed that the above symptoms were caused by hemodynamic insufficiency in the bilateral hemispheres with right-sided predominance, and a right-sided superficial temporal artery-middle cerebral artery bypass was performed. After the surgery, the patient was uneventful, but her obesity gradually worsened. Nineteen months after the bypass surgery, the MRI showed compensatory development of bilateral posterior choroidal arteries, and 29 months later, her weight was 87.9 kg and BMI was 33.5, further worsening her obesity, and the MRI showed an aneurysm formation on the right choroidal anastomosis. While considering the addition of revascularization surgery, as a result of obesity improvement (weight = 75 kg and BMI = 28.6) and blood pressure control as vascular risk management, the aneurysm spontaneously disappeared without bleeding on MRI five months after its confirmation. We report on the importance of vascular risk management in patients with MMD.

## Introduction

Moyamoya disease (MMD) is a cerebrovascular steno-occlusive disease characterized by progressive stenosis of the terminal portion of the internal carotid artery (ICA) and the formation of an abnormal network of dilated, fragile perforators in the base of the brain [1,2]. MMD can cause ischemic or hemorrhagic stroke, and well-recognized risks for the development of bleeding are choroidal anastomoses and peripheral aneurysms that form on these anastomoses [3-8]. It has been reported that neurosurgical procedures such as endovascular surgery and revascularization can reduce the risk of hemorrhage by occlusion or regression of these risk factors [1,2,6,7,9-11]. In this report, we describe an unusual case in which the aneurysm on the choroidal anastomosis formed more than two years after revascularization surgery in a patient with ischemic MMD spontaneously resolved with obesity improvement and the introduction of antihypertensive medication.

## Case presentation

**Figure 1:**
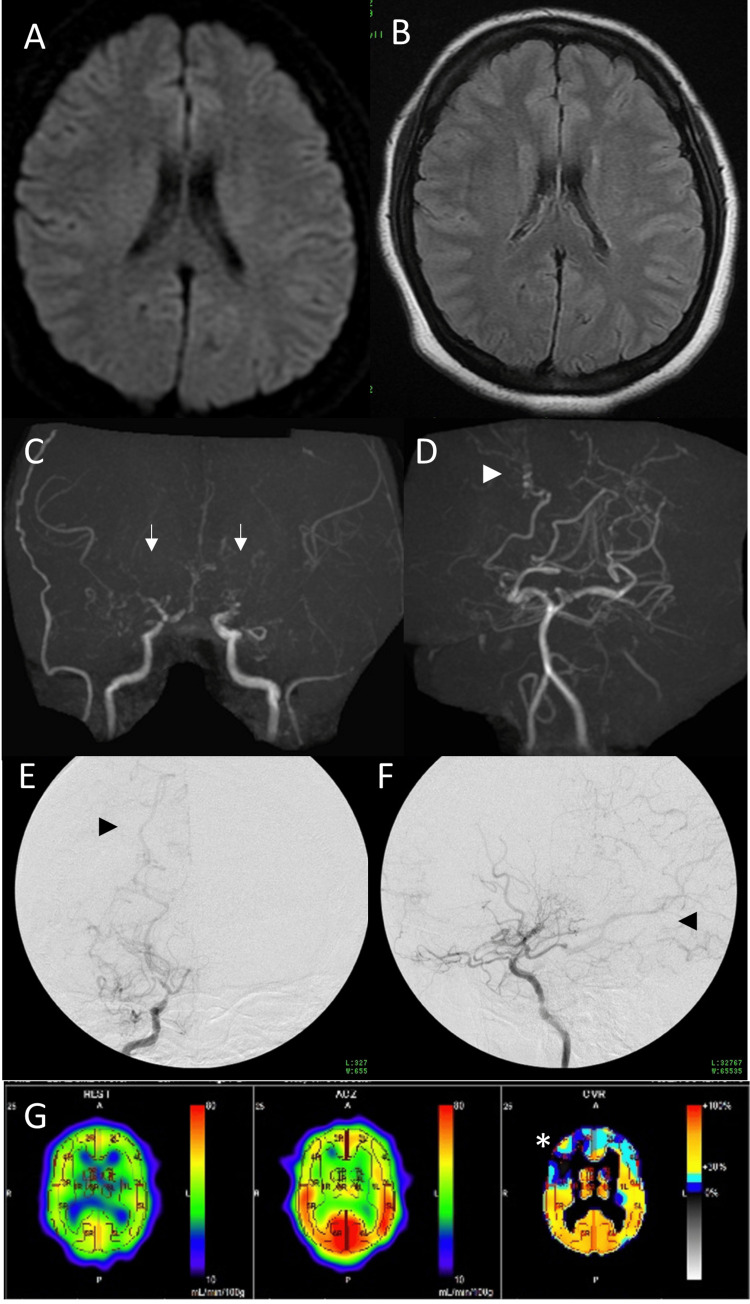
Preoperative imaging examination. A: Diffusion-weighted images showed no acute-phase infarct findings. B: Fluid-attenuated inversion recovery (FLAIR) images showed no abnormal findings; the periventricular flow void findings were unclear. C: Magnetic resonance angiography of the anterior circulation showed bilateral severe stenosis from the terminals of the internal carotid artery to the proximal portion of the anterior cerebral artery and middle cerebral artery, with nearby moyamoya vessels. D: Magnetic resonance angiography of the posterior circulation showed bilateral posterior cerebral arteries with compensatory development, but stenosis proximal to them was unclear. E and F: Preoperative digital subtraction angiography of the right internal carotid artery, anteroposterior (E) and lateral (F) views showed stenosis at the terminal of the internal carotid artery, moyamoya vessels, and a compensatory developed posterior cerebral artery (black arrowhead) are confirmed. G: Single photon emission computed tomography (SPECT) scan showed decreased resting cerebral blood flow and cerebrovascular reserve in the bilateral frontal lobes, predominantly on the right side.

A 24-year-old obese female (height = 162 cm, weight = 84 kg, and body mass index = 32.0) presented to our hospital with the chief complaint of transient dysarthria and left hemiparesis. On examination, she complained of a headache but had no other apparent neurological deficits. Blood samples showed dyslipidemia (Table 1), and head magnetic resonance imaging (MRI) showed no evidence of acute infarction (Figures 1A, 1B). However, magnetic resonance (MR) angiography showed significant stenosis from bilateral ICA terminals to the anterior and middle cerebral arteries (ACA/MCA) and moyamoya vessels at the base of the brain, leading to the diagnosis of MMD (Figures 1C, 1D). Digital subtraction angiography showed severe stenosis of bilateral ICA terminals, poor delineation of bilateral ACAs and MCAs, moyamoya vessels at the base of the brain, and collateral blood vessels from compensatorily dilated posterior cerebral artery (PCA) and dural arteries (Figures 1E, 1F). Single photon emission computed tomography (SPECT) showed decreased cerebral blood flow at rest and decreased cerebrovascular reserve in the bilateral frontal lobes, predominantly on the right side (Figure 1G).

**Table 1:** Pre- and postoperative dyslipidemia, weight, and blood pressure changes. HDL-C: high-density lipoprotein cholesterol; LDL-C: low-density lipoprotein cholesterol; BP: blood pressure.

Variable	Unit	Reference range	Preoperative	29 months after surgery	5 months after the aneurysm confirmation
Triglycerides	mg/dL	30-117	307	216	Not tested
HDL-C	mg/dL	48-103	42.6	45	Not tested
LDL-C	mg/dL	65-163	192	156	Not tested
Body weight	kg		84	87.9	75
BMI			32	33.5	28.6
				Before azilsartan administration	After azilsartan administration
Systolic BP	mmHg			128 (SD = 6)	106 (SD = 8)
Diastolic BP	mmHg			69 (SD = 12)	57 (SD = 14)

**Figure 2:**
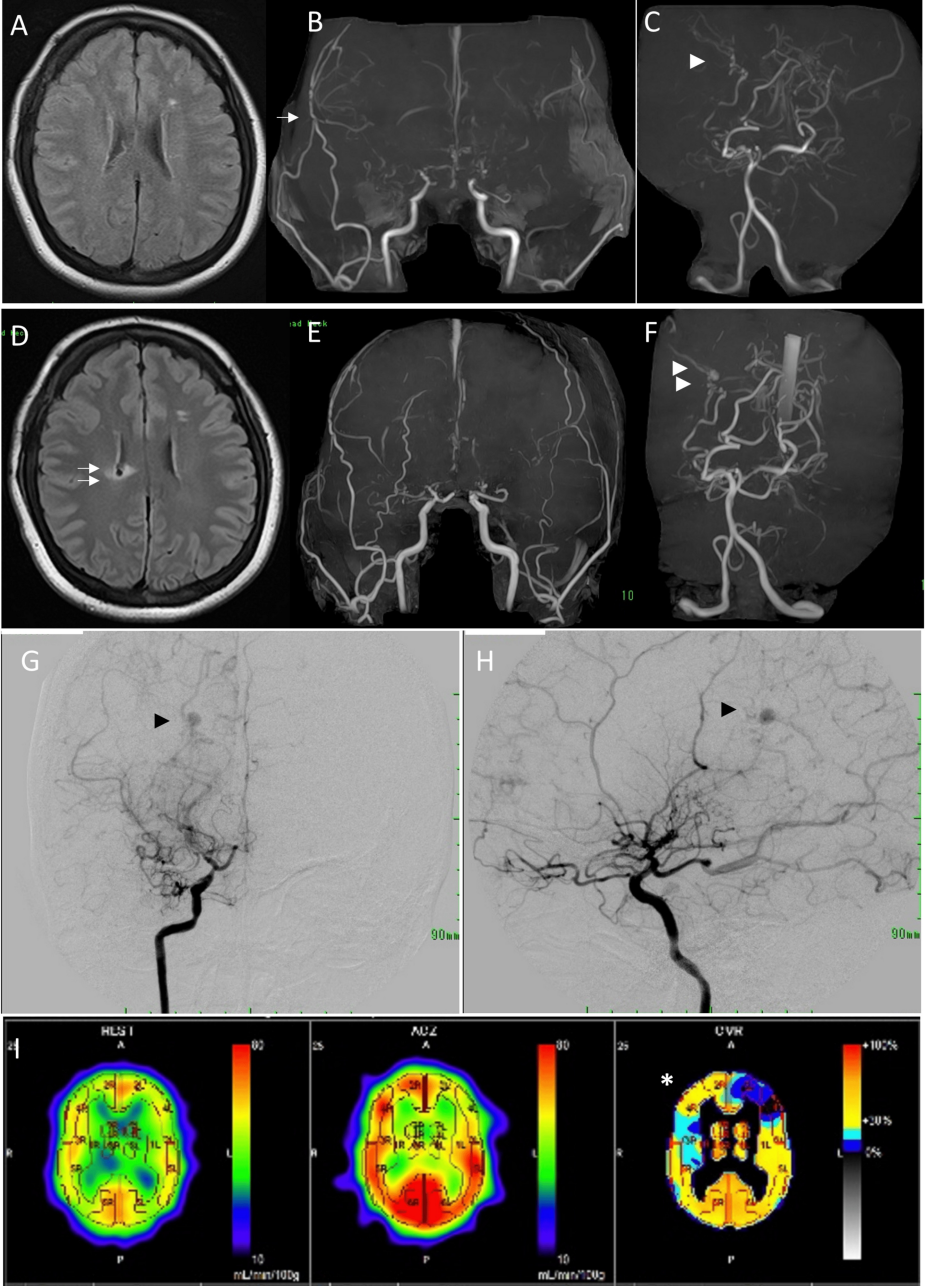
Postoperative follow-up imaging studies (A-C: 19 months after surgery; D-I: 29 months after surgery). A: Fluid-attenuated inversion recovery (FLAIR) images showed no clear flow void sign around the lateral ventricles. B: Magnetic resonance angiography of the anterior circulation showed that the superficial temporal artery to middle cerebral artery (STA-MCA) bypass was patent but not well developed (arrow). C: Magnetic resonance angiography of the posterior circulation showed the right-side developed and dilated lateral posterior choroidal artery as a collateral channel. D: A flow void signal with perilesional edema was observed along the right lateral ventricle (double arrow). E: Anterior circulation was unchanged. F: Aneurysm formation was observed in the peripheral portion of the right lateral posterior choroidal artery (asterisk). G and H: Digital subtraction angiography of the right internal carotid artery, anteroposterior (G) and lateral (H) images, revealed aneurysm formation (black arrowhead) at the peripheral portion of the developed posterior choroidal artery. I: Single photon emission computed tomography (SPECT) scan showed increased resting cerebral blood flow and improved cerebrovascular reserve in the right frontal lobe.

To prevent future strokes, she was administered dietary therapy for dyslipidemia and rosuvastatin 2.5 mg/day and underwent superficial temporal artery (STA)-MCA bypass surgery for the right hemisphere. Postoperatively, there were no ischemic or hemorrhagic complications, and a follow-up MRI showed that the patent bypass was not fully developed, but there were no recurrent strokes. At 19 months after bypass, compensatory dilatation of the bilateral posterior choroidal arteries (PChAs) became prominent on MRI (Figures 2A-2C). At 29 months, the SPECT scan showed improvement in the right hemispheric hemodynamic insufficiency (HI) (Figure 2I). However, an aneurysm was formed in the peripheral portion of the right PChA on MRI (Figures 2D-2H), at which time her obesity became even more pronounced (weight = 87.9 kg and BMI = 33.5).

The plan was to strictly manage her obesity and blood pressure (BP) while considering the addition of bypass procedures. Azilsartan 20 mg/day was administrated, and the patient was started on a self-imposed diet and exercise therapy such as aerobics without any specific professional assistance for obesity improvement. The patient had no episodes of suspected cerebral hemorrhage or infarction, and by the fifth month after confirmation of the aneurysm, her obesity had improved to a weight of 75 kg and BMI of 28.6, and her mean BP decreased from 128/69 mmHg to 106/57 mmHg after the introduction of azilsartan (Table 1).

**Figure 3:**
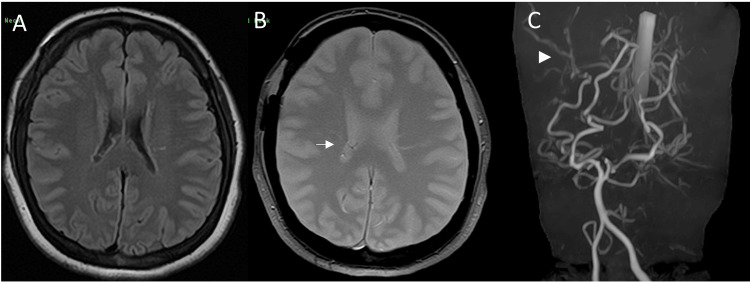
MRI scan five months after the aneurysm confirmation. A: The flow void signal with edema around the right lateral ventricle had disappeared. B: The T2 star image also showed no hemosiderin deposition. C: The peripheral aneurysm of the right lateral posterior choroidal artery had disappeared.

MRI scan of the head showed that compensatory development of bilateral PChAs was still present, but the peripheral aneurysm of the right PChA had disappeared (Figures 3A, 3C). There were no hemosiderin deposits on the T2 star-weighted image (Figure 3B), and the aneurysm was considered to have spontaneously regressed without bleeding.

The patient continued the diet and exercise therapy, and nine months had passed since the aneurysm was confirmed to have regressed, but there was no recurrence of the aneurysm. However, the dangerous collateral vessels of the choroidal anastomosis remained, and the patient was still at high risk of developing hemorrhage. We continue to consider the indication for revascularization surgery.

## Discussion

MMD is a cerebrovascular steno-occlusive condition characterized by progressive stenosis of the terminal portion of the ICAs and the formation of an abnormal network of dilated, fragile perforators at the base of the brain. The prevalence of MMD is estimated to be 0.1 per 100,000 people worldwide but is higher in East Asian countries, especially Japan (3.16 to 10.5 per 100,000) and Korea (6.3 to 16.1 per 100,000). MMD presents with a bimodal age distribution at approximately 10 years and 30 to 50 years [1,2]. No prospective randomized controlled trials are addressing surgical revascularization for MMD patients with ischemic stroke. However, the 2021 guidelines of the Japanese Stroke Association report that surgical revascularization for MMD patients with ischemic stroke reduces the frequency of transient ischemic attacks and the risk of stroke and improves the long-term prognosis for postoperative activities of daily living and neurocognitive function [11]. The Japanese Adult Moyamoya Disease Trial, a randomized trial of surgical revascularization (bilateral direct bypass) versus medical therapy for hemorrhagic MMD, also showed a reduction in rebleeding with surgery (2.7%/year versus 7.6%/year; P = 0.04) [10]. Its prespecified analysis reported a higher risk of rebleeding in the posterior hemorrhage group, defined as bleeding originating from the PCA or choroidal artery, and a more significant preventive effect of surgical revascularization on rebleeding [12].

Although choroidal anastomosis is considered a potential risk for rebleeding [3-5,8,12], aneurysms developing on the choroidal anastomosis are also a critical bleeding risk [6,7,13,14]. Once this bleeds, conservative treatment increases the likelihood of rebleeding, resulting in an outcome of death or permanent disability, so urgent treatment such as endovascular or revascularization surgery should be considered [6,7,13]. Local hemodynamic stress is regarded as a trigger for aneurysm formation [13-15], which may be true as aneurysms on the choroidal anastomosis disappear after revascularization surgery, reducing the risk of rebleeding [6,13]. On the other hand, there have been reports of spontaneous regression of once-bleeding aneurysms without the need for these treatments [14,16]. However, we presented here an interesting case of an aneurysm on the choroidal anastomosis that occurred 29 months after STA-MCA bypass surgery and spontaneously resolved with weight and BP management, without bleeding, and without requiring endovascular surgery or additional revascularization procedures. In the present case, why did the aneurysm develop on the choroidal anastomosis despite the preceding bypass surgery? The fact that the aneurysm disappeared with weight loss and BP control suggests that obesity and associated BP changes may have been involved to some extent.

On the other hand, the STA-MCA bypass was poorly developed, suggesting that endogenous collateral channels such as choroidal anastomosis improved HI, resulting in aneurysm formation due to excessive hemodynamic stress on the PChA. The targeted bypass strategy that selects as a recipient the cortical arteries around the brain surface where the medullary arteries, which serve as collateral pathways from the PChA, originate has been reported to prevent bleeding in MMD effectively [17]. If the peripheral aneurysm in the case presented here had developed hemorrhage, endovascular surgery to prevent rebleeding would be a reasonable indication, considering the risk of ischemic complications. However, if the aneurysm has not yet ruptured, endovascular surgery is technically challenging, and ischemic complications from this procedure are unacceptable. A targeted bypass strategy is very reasonable, but in this case, the STA is already being used as a donor artery, the auricular artery is poorly developed, and if the occipital artery (OA) is used as a donor, it is unlikely to reach the target site mentioned above, even if bypass surgery is added. Considering that bypass surgery for the contralateral hemisphere may be necessary, we are hesitant to harvest the contralateral STA as a graft or donor artery. Therefore, we carefully consider targeted bypass using the STA trunk of the poorly developed STA-MCA bypass or OA as a donor and the radial artery as an interposed graft.

The relationship between obesity and hypertension is widely recognized as a result of the interrelated mechanisms induced by obesity, including activation of the sympathetic nervous system, the renin-angiotensin-aldosterone system, and abnormal renal function. Weight loss may also ameliorate obesity-related hypertension and reduce cardiovascular risk [18,19]. As for the vascular risk profile of MMD patients undergoing revascularization, the age- and sex-adjusted prevalence of hypertension was increased, especially in women aged 30-44 years and in male and female patients aged 45-64 years, but was similar to the general population for obesity [20]. However, another report has shown that the relationship between MMD and obesity is associated with the expression of the ring finger protein 213 (RNF213) gene and is particularly affected via tumor necrosis factor-alpha-mediated inflammation and insulin resistance in adipocytes. In obese patients, high expression of RNF213 may serve as an adaptive mechanism to suppress adipogenesis. These findings provide important clues to understanding the pathophysiology of MMD and suggest that obesity may influence the development and progression of MMD [21]. Rosuvastatin and azilsartan may prevent the rupture of unruptured cerebral aneurysms [22], but there are no reports of aneurysm resolution after taking these drugs. In this case, there was no family history of obesity, and the reduction of vascular risk, such as weight loss, may have improved the above pathological mechanism and reduced the hemodynamic stress on the choroidal anastomosis, resulting in spontaneous resolution of the aneurysm on its collateral vessels. Vascular risk management is essential for patients with MMD, even after revascularization surgery.

## Conclusions

We reported a rare case in which a peripheral aneurysm of the PChA regressed after conservative treatment, with improvement in obesity and the introduction of an antihypertensive drug. Choroidal anastomosis and aneurysms occurring on the collateral vessels are important risk factors for bleeding, and the efficacy of revascularization surgery is well known. This report demonstrates the importance of neurosurgical procedures and vascular risk management in reducing the risk of hemorrhage. Of course, in this case, the aneurysm disappeared; however, the dangerous choroidal anastomosis remains, and the risk of developing hemorrhage remains high, so the indication for revascularization surgery continues to be under consideration.
